# Hot Deformation Behavior and Hot Processing Map Analysis of Q420DR Low-Temperature Pressure Vessel Steel

**DOI:** 10.3390/ma19194194

**Published:** 2026-09-30

**Authors:** Xianpeng Zhao, Changlin Liu, Yuan Gao, Mengqi Wang, Kai Lv, Yuzhuo Zhao, Rungang Wang, Ling Wang, Xianming Zhao

**Affiliations:** 1 State Key Laboratory of Digital Steel, Northeastern University, Shenyang 110819, China; 2390065@stu.neu.edu.cn (X.Z.); 2470539@stu.neu.edu.cn (M.W.); lvkai@mails.neu.edu.cn (K.L.); zhaoyz10@mails.neu.edu.cn (Y.Z.); 2 Shandong Iron & Steel Group Rizhao Co., Ltd., Lin Gang Road No. 1, Donggang District, Rizhao 276800, China; ustbwrg@163.com; 3 School of Materials Engineering, Inner Mongolia University of Science and Technology, Baotou 014010, China; quwei@imust.edu.cn; 4 Jingmen Hongtu Special Aircraft Manufacturing Co., Ltd., No. 16 Yingchun Avenue, Duodao District, Jingmen 448124, China; ling.wang@enricgroup.com

**Keywords:** Q420DR steel, hot deformation behavior, Zener–Hollomon parameter, hot processing map

## Abstract

Nominally isothermal compression tests were performed on Q420DR low-temperature pressure-vessel steel at 900–1150 °C and strain rates of 0.05–10 s^−1^, with a target true strain of 0.7. The effects of temperature and strain rate on flow behavior were investigated. Flow stress increased with decreasing temperature and increasing strain rate, while higher temperatures and lower strain rates favored more pronounced flow softening. A peak-stress-based Arrhenius-type constitutive relationship was established, yielding an apparent hot-deformation activation energy of 385.987 kJ·mol^−1^. Processing maps based on the dynamic materials model were constructed using experimental flow stresses at fixed true strains of 0.1–0.6. At a true strain of 0.6, relatively high power-dissipation efficiencies combined with positive instability parameters indicated a potentially favorable processing domain at approximately 1050–1100 °C under low strain rate conditions. Predicted flow instability was mainly concentrated at low temperatures and relatively low strain rates. The distributions of power-dissipation efficiency and instability boundaries varied with strain, indicating that processing conditions should be assessed over the deformation path. These findings provide preliminary guidance for hot working under the investigated single-pass compression conditions.

## 1. Introduction

Low-alloy low-temperature pressure vessel steels are indispensable structural materials for cryogenic chemical equipment, liquefied gas storage devices, and high-pressure energy engineering systems due to their superior strength, low-temperature toughness and structural stability [1,2,3,4]. As a newly standardized low-temperature pressure vessel steel specified in GB/T 713.3-2023 [5], Q420DR steel is specially developed for the manufacturing of low-temperature mobile storage tanks that serve liquefied petroleum gas (LPG, commercial propane and butane mixtures), showing great application prospects in domestic cryogenic equipment localization and replacing the conventional European P420NL fine-grained low-temperature vessel steel [6,7,8]. To satisfy the rigorous service safety requirements of low-temperature and high-pressure working conditions, Q420DR steel imposes strict mechanical property specifications: the yield strength is no less than 420 MPa, the tensile strength ranges from 590 MPa to 720 MPa, the elongation is greater than 18%, the Charpy impact energy at −40 °C exceeds 60 J, and the lateral expansion of impact specimens is required to be higher than 0.53 mm [9]. In addition, post-weld heat treatment (PWHT) is mandatory for engineering applications, which is implemented at 580–610 °C with a holding time of 30–60 min to eliminate welding residual stress and improve structural safety [10,11,12]. Benefiting from its well-matched mechanical performance and standardized heat treatment system, Q420DR has been gradually popularized in the fabrication of large-scale cryogenic pressure components [13,14,15]. However, as a newly released steel grade, its fundamental hot deformation theory and processing regulation system are still incomplete. The coupling relationship between hot deformation parameters, microstructure evolution and mechanical properties has not been systematically clarified, which seriously restricts its high-quality industrial hot forming and large-scale engineering promotion.

Hot deformation behavior determines the microstructural evolution and final service performance of metallic structural steels [16,17,18]. Hot rolling and hot forging are the core forming processes for pressure vessel steel components, and unreasonable deformation temperature and strain rate will easily cause dynamic strain aging, local shear band concentration and microcrack initiation, resulting in deteriorated low-temperature toughness and unstable mechanical properties of finished products. Therefore, systematically exploring the hot flow behavior, deformation resistance mechanism and stable processing range is the premise to realize precise control of hot forming quality for pressure vessel steels.

The hot processing map based on the dynamic material model (DMM) has become a mainstream and effective method to evaluate the hot workability of metal materials [19,20,21]. It can intuitively distinguish the stable deformation domain dominated by dynamic recrystallization and dynamic recovery and the unstable deformation domain prone to plastic defects, realizing the quantitative optimization of hot processing parameters. Previous studies have investigated the constitutive behaviour of various steels at elevated temperatures [22,23,24]. More recently, Liu et al. [25] studied the hot deformation behaviour of Q420 steel by combining constitutive modelling, dynamic recrystallization modelling and microstructural characterization. There are few systematic reports on the hot deformation behavior, constitutive relationship and hot workability characteristics of the new Q420DR steel grade, and its optimal hot processing window and instability mechanism have not been clarified, resulting in excessive empirical dependence in actual production.

In this work, nominally isothermal hot compression tests were performed on Q420DR low-temperature pressure-vessel steel using a DIL805 deformation dilatometer. The effects of deformation temperature and strain rate on flow behavior were investigated. An Arrhenius-type constitutive relationship was established using peak-stress data, and the apparent hot-deformation activation energy was determined. Processing maps were constructed at true strains of 0.1–0.6 using the corresponding fixed-strain flow stresses to evaluate the evolution of power-dissipation efficiency and predicted flow instability. The contribution of this study is the combined characterization of the peak-stress response and strain-dependent processing-map evolution of Q420DR steel, providing material-specific data for identifying candidate hot-working conditions.

## 2. Materials and Methods

The experimental material adopted in this work is a commercial Q420DR low-temperature pressure vessel steel supplied by industrial manufacturers. The chemical composition (wt.%) of the tested steel is listed in Table 1.

Single-pass isothermal hot compression tests were conducted on a DIL805 deformation dilatometer (TA Instruments, Hüllhorst, Germany) to investigate the hot deformation behavior of Q420DR steel. Cylindrical specimens with a diameter of 5 mm and a height of 10 mm were mechanically polished using abrasive papers before testing. Tantalum sheets and lubricant were used between the specimen end faces and the anvils to reduce interfacial friction. The DIL805 dilatometer adopts a resistance heating system and high-vacuum test chamber, which can effectively avoid surface oxidation during high-temperature deformation. A thermocouple was welded on the specimen surface to realize real-time and accurate temperature monitoring throughout the entire test process. Benefiting from the synergistic control of resistance heating and thermocouple feedback, the temperature control accuracy of the equipment reaches ±1 °C. The tests were conducted under nominally isothermal conditions. Although specimen temperature was monitored using a surface-welded thermocouple, the transient internal adiabatic temperature rise during deformation at 10 s^−1^ was not independently quantified or corrected.

**Figure 1:**
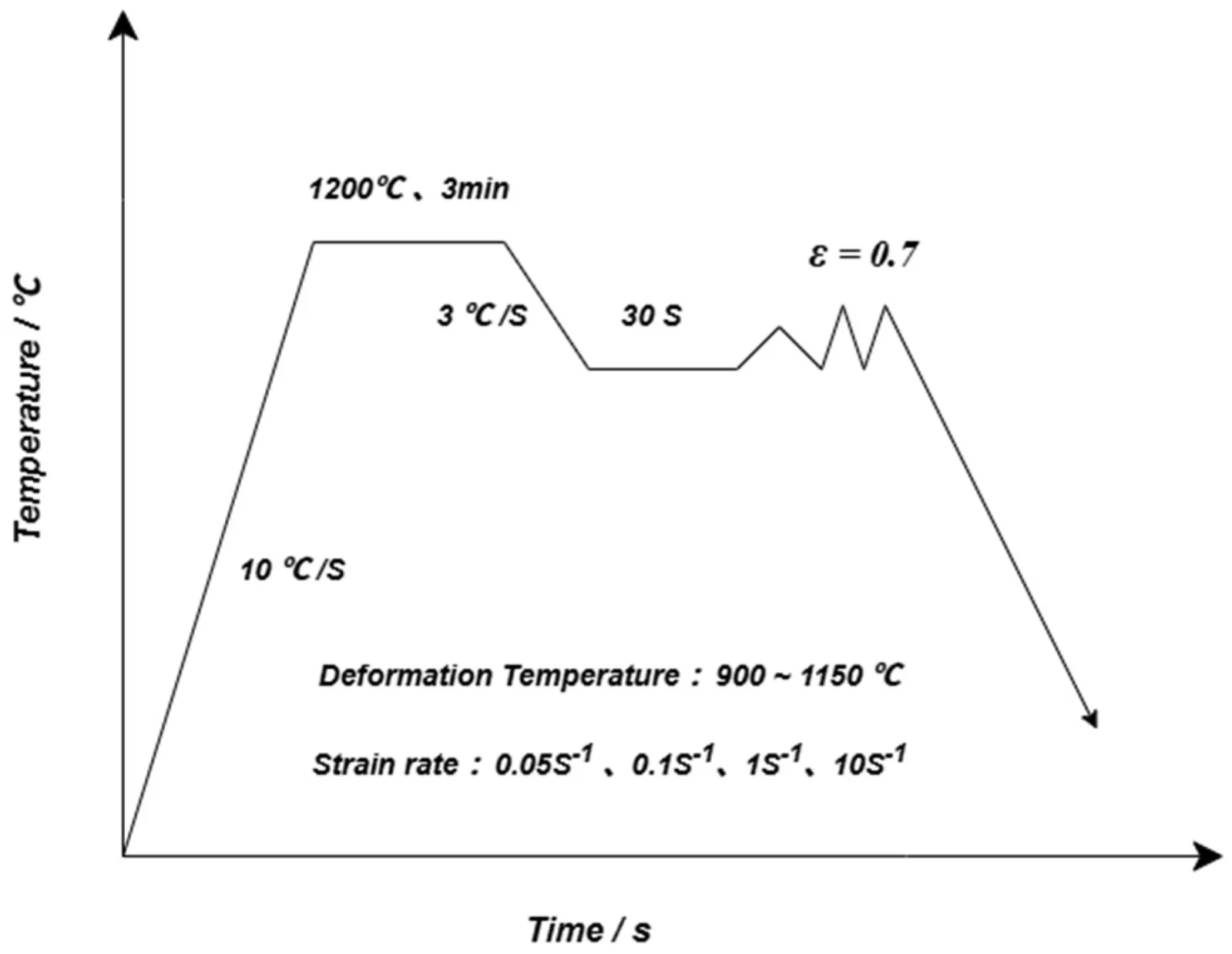
Thermal-mechanical procedure of single-pass compression of Q420DR steel.

The detailed thermomechanical test procedure is illustrated in Figure 1. Prior to deformation, the specimen was heated to 1200 °C at a heating rate of 10 °C/s and held for 3 min to acquire a homogeneous and stable initial microstructure. Subsequently, the specimen was cooled to target deformation temperatures of 900 °C, 950 °C, 1000 °C, 1050 °C, 1100 °C, and 1150 °C at a cooling rate of 3 °C/s, followed by an isothermal holding for 30 s to eliminate internal temperature gradients. Single-pass hot compression was performed at strain rates of 0.05, 0.1, 1, and 10 s^−1^, with a target true strain of 0.7. Immediately after compression, the specimens were water-quenched to room temperature. For processing-map construction, flow stresses at true strains of 0.1, 0.2, 0.3, 0.4, 0.5, and 0.6 were extracted from the valid loading portions of the curves. This common strain range was selected to ensure comparability among all test conditions and to avoid the influence of deceleration and unloading near the end of compression.

## 3. Results and Discussion

### 3.1. Flow Stress Curves

**Figure 2:**
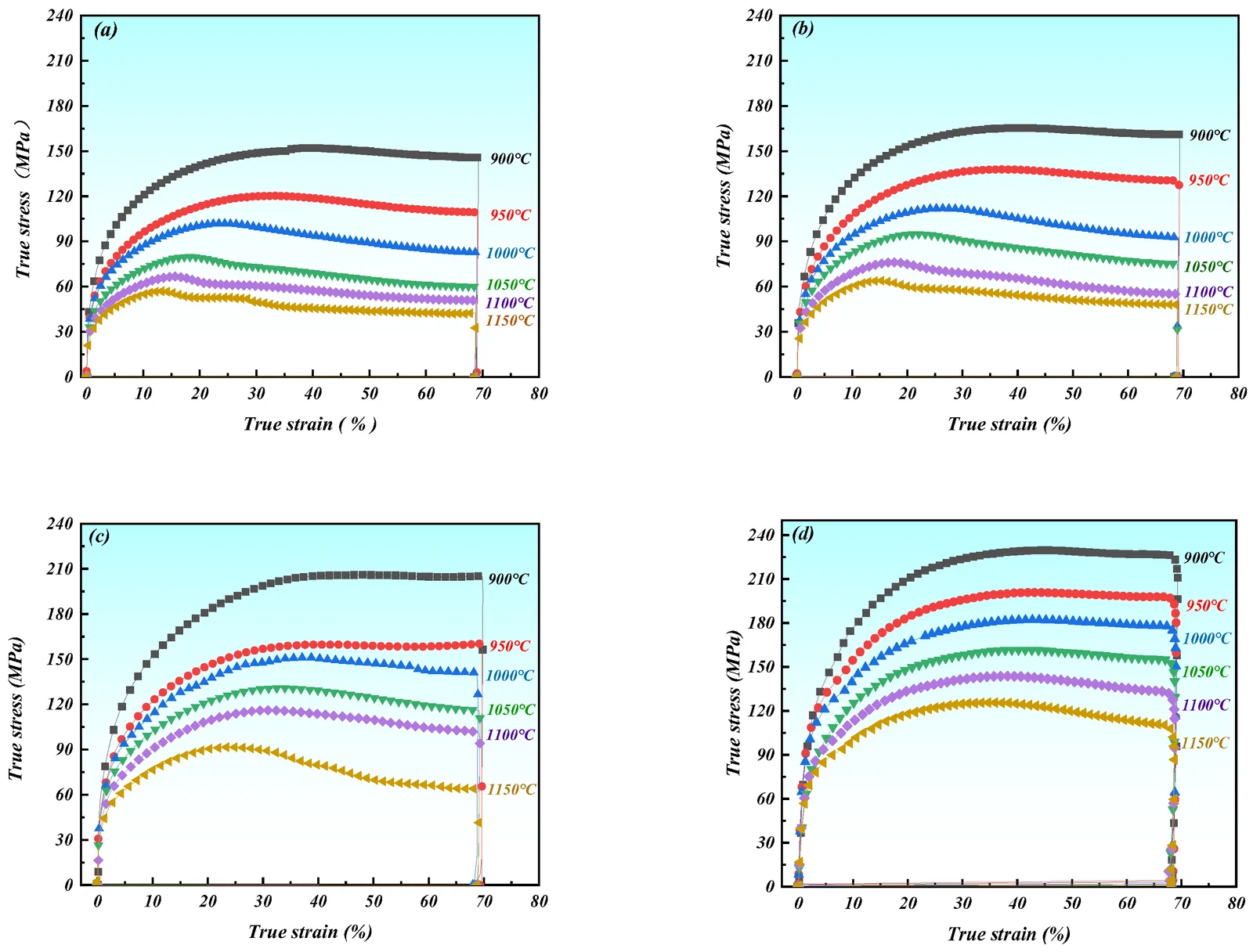
True stress-true strain curves of Q420DR steel at different strain rates. (**a**) $\dot{\varepsilon }$ = 0.05 s^−1^; (**b**) $\dot{\varepsilon }$ = 0.1 s^−1^; (**c**) $\dot{\varepsilon }$ = 1 s^−1^; (**d**) $\dot{\varepsilon }$ = 10 s^−1^.

Isothermal hot compression tests were carried out at temperatures of 900–1150 °C and strain rates of 0.05–10 s^−1^ to obtain the true stress–true strain curves of Q420DR steel. The corresponding flow curves are presented in Figure 2, in which Figure 2a–d represent the deformation characteristics at strain rates of 0.05 s^−1^, 0.1 s^−1^, 1 s^−1^, and 10 s^−1^, respectively. The evolutionary characteristics of the curves intuitively reflect the coupled effects of temperature and strain rate on the hot deformation behavior of Q420DR steel.

Figure 2a shows the flow stress curves obtained at an ultra-low strain rate of 0.05 s^−1^. Sufficient deformation duration enables adequate atomic diffusion, dislocation slip, and grain boundary migration under such conditions. All curves exhibit a typical evolutionary feature consisting of work hardening, peak softening, and steady-state flow. In the initial deformation stage, rapid dislocation multiplication induces intense work hardening, leading to a sharp increase in flow stress. After reaching the peak, the flow stress decreases as dynamic softening outweighs work hardening. Dynamic recovery (DRV) and dynamic recrystallization (DRX) may contribute to this softening. With the increase in deformation temperature, the peak stress decreases significantly and the flow stress approaches a more stable level. A prominent softening effect is observed at 1050–1150 °C, indicating that the dynamic softening mechanism of Q420DR steel can be fully activated under ultra-low strain rate and high-temperature conditions. In contrast, relatively high peak stress and insufficient softening are detected at 900–950 °C, indicating less pronounced flow softening at low temperatures.

Figure 2b displays the flow stress curves at a low strain rate of 0.1 s^−1^. Compared with 0.05 s^−1^, the deformation period is slightly shortened, and the overall variation trend of the curves remains consistent, while the overall flow stress level increases moderately. In the low-temperature range of 900–950 °C, the work-hardening effect is enhanced, the peak stress increases, and the critical strain corresponding to the peak shifts to a higher strain value, demonstrating delayed and weakened dynamic softening. In the high-temperature range of 1050–1150 °C, the flow stress approaches a relatively steady level after reaching the peak. A good balance between work hardening and dynamic softening is obtained, which reflects excellent hot deformation compatibility and matches the optimal low strain rate processing window.

Figure 2c illustrates the flow stress curves at a medium-high strain rate of 1 s^−1^. The shortened deformation time significantly delays the occurrence of DRV and DRX and restricts the dynamic softening behavior. The flow stress values over the entire temperature range are considerably higher than those at low strain rates, with prominent peak characteristics and a narrowed post-peak softening range, and no distinct steady-state softening stage is observed. At 900–950 °C, work hardening dominates the entire deformation process without effective dynamic softening, resulting in high flow stress and large deformation resistance, which easily induces inhomogeneous deformation. Even at high temperatures, dynamic recrystallization cannot develop sufficiently, and only slight stress relaxation occurs, leading to a significant deterioration in hot deformation stability.

Figure 2d presents the flow stress curves at the highest tested strain rate of 10 s^−1^. The instantaneous deformation process causes rapid accumulation and entanglement of dislocations, making work hardening absolutely dominant. The maximum peak stress is achieved under this condition, accompanied by a further increase in critical strain. In the low-temperature range of 900–950 °C, the curves maintain a continuous hardening trend without obvious softening features, implying a high risk of microstructural defect accumulation. At 10 s^−1^, rapid dislocation accumulation produces a high flow-stress level and delays the establishment of dynamic softening. It should also be noted that the short deformation duration may cause partial conversion of plastic work into heat. Therefore, any apparent high-strain softening at 10 s^−1^ may reflect the combined effects of microstructural evolution and deformation heating.

Comprehensive analysis of the four groups of flow curves indicates that the hot deformation behavior of Q420DR steel exhibits strong coupling sensitivity to temperature and strain rate. More pronounced flow softening is observed at low strain rates (0.05–0.1 s^−1^) and relatively high temperatures (1050–1150 °C), suggesting an increased contribution from dynamic softening relative to work hardening. In contrast, high strain rates combined with low temperatures inhibit dynamic softening and induce excessive work hardening, resulting in large deformation resistance and poor deformation stability. The clarified flow behavior provides a reliable experimental basis for the calculation of hot deformation activation energy, establishment of constitutive equations, and optimization of the hot processing window of Q420DR steel. Although tantalum sheets and lubricant were used, residual interfacial friction cannot be completely excluded, particularly at larger strains. The associated barreling constraint may lead to some overestimation of the measured flow stress during the later stages of compression.

### 3.2. Peak-Stress-Based Arrhenius Constitutive Relationship

To characterize the interplay among hot-deformation activation energy, forming temperature and applied strain rate under hot-compression loading, constitutive modelling is an indispensable tool for describing the high-temperature flow behavior of metallic alloys [26,27,28,29]. Among multiple constitutive frameworks reported in existing literature, the hyperbolic-sine Arrhenius model originally developed by Sellars and Tegart exhibits strong adaptability for capturing hot-flow characteristics over broad deformation conditions for most metallic materials [30]. The mathematical form of this physical model is given as [30]:

(1) $$\dot{\varepsilon }=A{\left\{\sinh(\alpha \sigma_{p})\right\}}^{n}\exp\left(-Q_{d}/\mathrm{RT}\right)$$

where $\dot{\varepsilon }$ denotes strain rate (s^−1^); *α* is a material-dependent stress constant (MPa^−1^); σ*_p_* is peak flow stress extracted from hot-compression experimental curves (MPa); *n* stands for the stress exponent that reflects the material’s intrinsic strain rate sensitivity; *Q_d_* is hot-deformation activation energy (kJ·mol^−1^); *R* corresponds to the universal gas constant, which is set as 8.314 J/(mol·K); *T* is absolute deformation temperature (K); and *A* is the pre-exponential material constant.

For low-stress and high-stress limiting scenarios, Equation (1) can be simplified into two alternative expressions. Equation (2) applies to low-stress circumstances satisfying levels (i.e., ασ < 0.8), while Equation (3) is valid for high-stress states where (i.e., ασ > 1.2). Comparatively, Equation (1) with a hyperbolic-sine function works across the full stress range investigated in this study. In these formulas, *A*_1_, *A*_2_, *n*_1_, and *β* are constants determined from experimental data, and the correlation between the two stress constants follows *α* = *β*/*n*_1_.

(2) $$\dot{\varepsilon }=A\sigma^{n_{1}}\exp\left(-Q_{d}/\mathrm{RT}\right)$$

(3) $$\dot{\varepsilon }=\mathrm{Aexp}(\beta \sigma_{p})\exp\left(-Q_{d}/\mathrm{RT}\right)$$

A natural logarithm transformation is performed on both sides of Equations (2) and (3), yielding Equations (4) and (5):

(4) $$\ln\dot{\varepsilon }=n_{1}\ln\sigma_{p}+\ln A_{1}-Q_{d}/\mathrm{RT}$$

(5) $$\ln\dot{\varepsilon }=\beta \sigma_{p}+\ln A_{2}-Q_{d}/\mathrm{RT}$$

**Figure 3:**
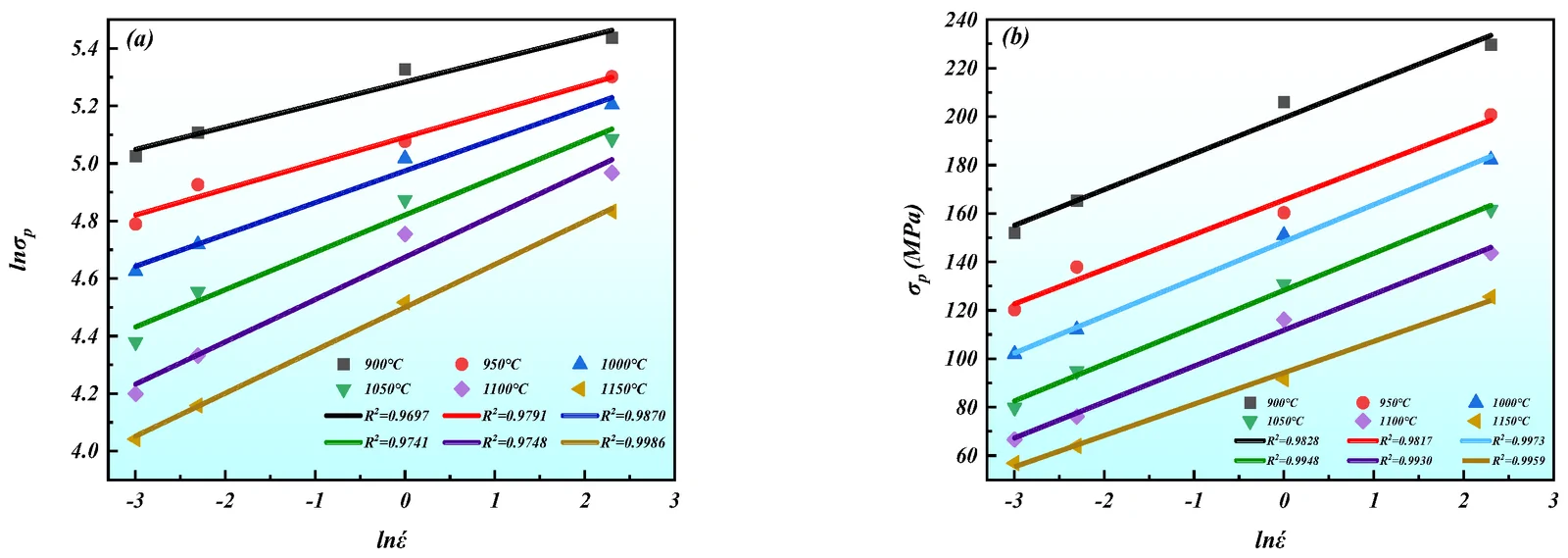
Correlation between peak stress and strain rate for Q420DR steel: (**a**) $\ln\dot{\varepsilon }-\ln\sigma_{p}$; (**b**) $\ln\dot{\varepsilon }-\sigma_{p}$.

From the transformed formulas, linear correlations can be established between $\ln\dot{\varepsilon }-\sigma_{p}$, as well as between $\ln\dot{\varepsilon }-\ln\sigma_{p}$. Using peak-stress datasets measured under diverse thermomechanical conditions, linear regression was implemented to acquire the slopes of fitting lines. The fitted results are visualized in Figure 3a,b. The values of *n*_1_ and *β* were obtained by computing reciprocals of regression slopes and further averaging these outputs, giving *n*_1_ = 8.823 and β = 0.06815. On this basis, the stress constant *α* = *β*/*n*_1_ was calculated to be 0.007725 (MPa^−1^) .

Taking the logarithm of both sides of Equation (1), the rearranged form is written as Equation (6).

(6) $$\ln\dot{\varepsilon }=n_{2}\ln\left\{\sinh(\alpha \sigma_{p})\right\}+\mathrm{lnA}-Q_{d}/\mathrm{RT}$$

**Figure 4:**
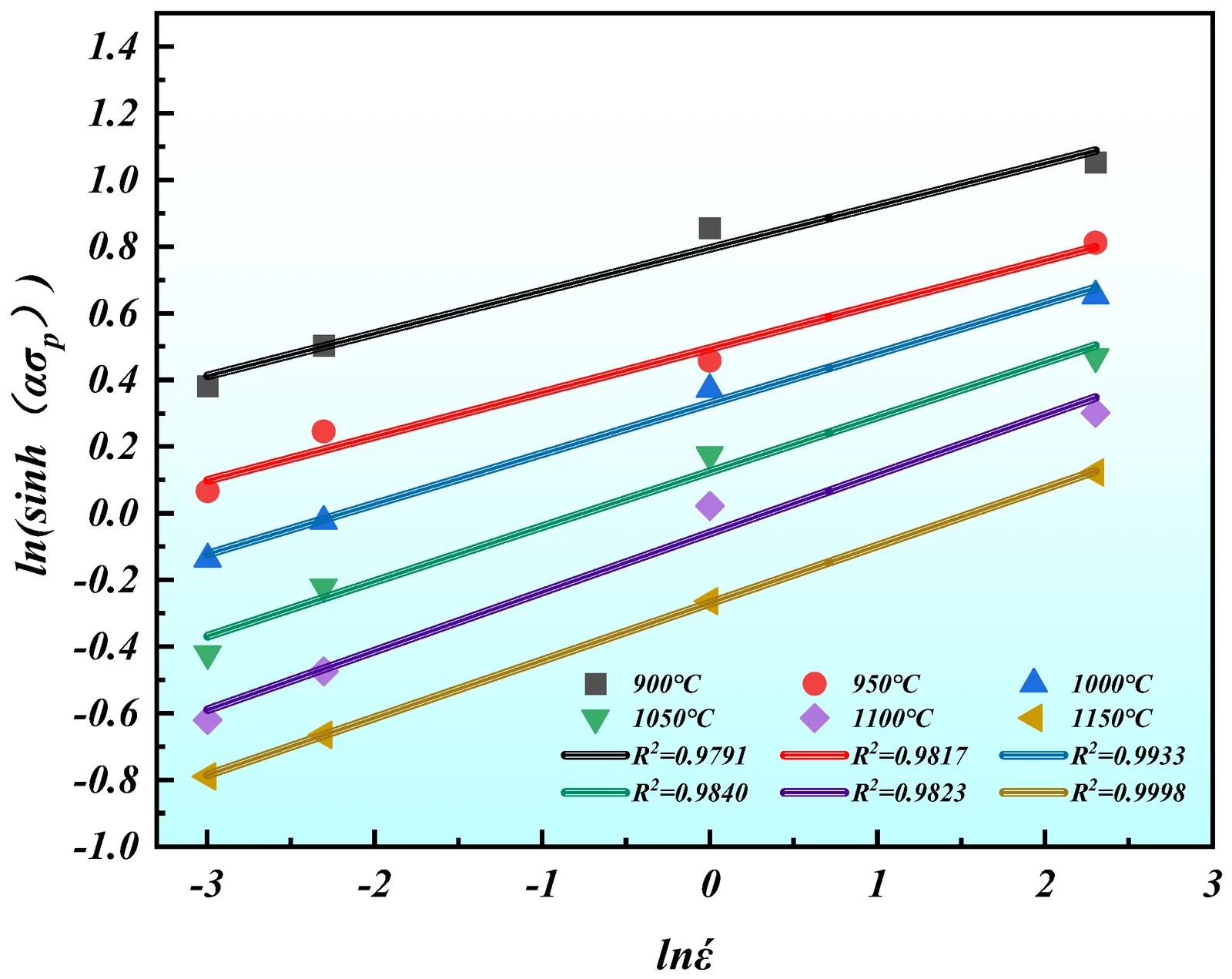
Relationship between lnsinh(*ασ_p_*) and $\ln\dot{\varepsilon }$ of Q420DR steel.

A favorable linear trend is observed between lnsinh(*ασ_p_*) and $\ln\dot{\varepsilon }$, as demonstrated in Figure 4. The stress exponent *n*_2_ was derived from the mean reciprocal value of regression slopes, and the calculated *n*_2_ equals 6.5053.

Partial derivative operation with respect to (1/*T*) was carried out for Equation (6), which produces Equation (7):

(7) $$Q_{d}=R\left|\partial \ln\dot{\varepsilon }/\partial \ln\left\{\sinh(\alpha \sigma_{p})\right\}\right|.\left|\partial \left\{\sinh(\alpha \sigma_{p})\right\}/\left(\partial 1000/Τ\right)\right|$$

**Figure 5:**
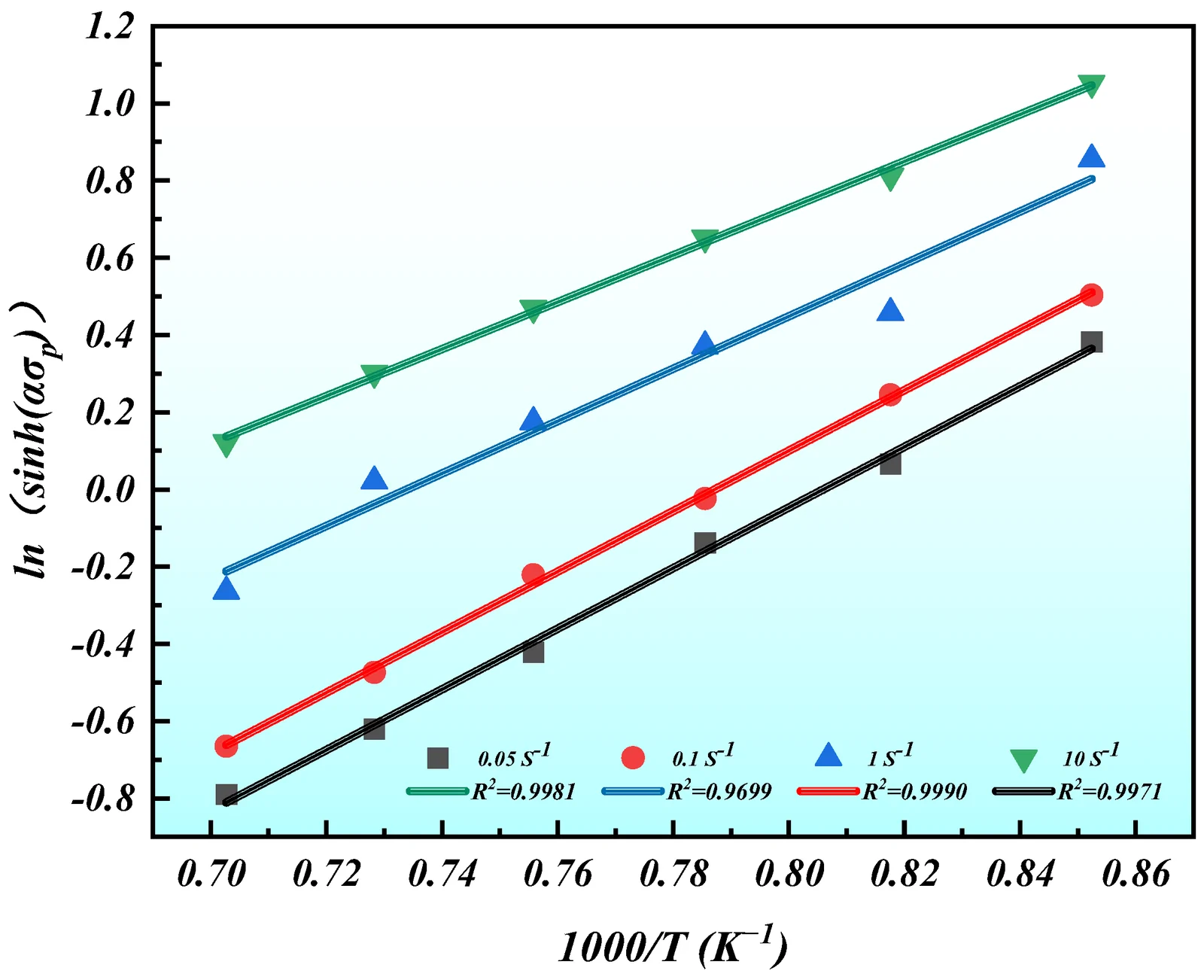
Relationship between lnsinh(*ασ_p_*) and 1000/*T* (*K*^−1^) of Q420DR steel.

The dataset of lnsinh(*ασ_p_*) was plotted against 1000/*T*, as presented in Figure 5. Linear regression delivers an averaged slope *k*_(average)_ = 7.137. Substituting the gas constant value into the formula *Q* = 1000 *Rn*_2_*k*, the hot-deformation activation energy for Q420DR steel was finally determined as *Q_d_* = 385.987 kJ·mol^−1^.

The calculated value of 385.987 kJ mol^−1^ should be interpreted as an apparent hot-deformation activation energy rather than the energy barrier of a single diffusion process. Q420DR steel contains Mn and Ni together with the microalloying elements Nb, V and Ti. Solute atoms and possible microalloying-element carbonitrides can impede dislocation motion and grain-boundary migration, thereby increasing the temperature sensitivity of dynamic recovery and recrystallization. Moreover, the activation energy was obtained by globally fitting peak stresses over a wide temperature range, within which the relative contributions of work hardening, recovery and recrystallization may vary. Consequently, the calculated value represents the combined response of multiple thermally activated processes. Because precipitation behavior was not directly characterized in the present study, the contribution of individual precipitate species cannot be quantified and should be investigated separately in future work.

Zener and Hollomon proposed a composite physical parameter to couple temperature and strain rate effects during high-temperature deformation, known as the Zener–Hollomon (*Z*) parameter. Its mathematical definition is shown in Equation (8):

(8) $$Z=\dot{\varepsilon }\exp\left(Q_{d}/\mathrm{RT}\right)=A{\left\{\sinh(\alpha \sigma_{p})\right\}}^{n}$$

Taking the logarithm of both sides of Equation (8) further yields:

(9) lnZ = *n*ln{sinh(*ασ*_p_)} + lnA

By combining Equations (1) and (8), the following can be obtained:

(10) $$\sigma =\left(1/\alpha \right)\ln\left\{{\left(Z/A\right)}^{1/n}+{\left[{\left(Z/A\right)}^{2/n}+1\right]}^{1/2}\right\}$$

**Figure 6:**
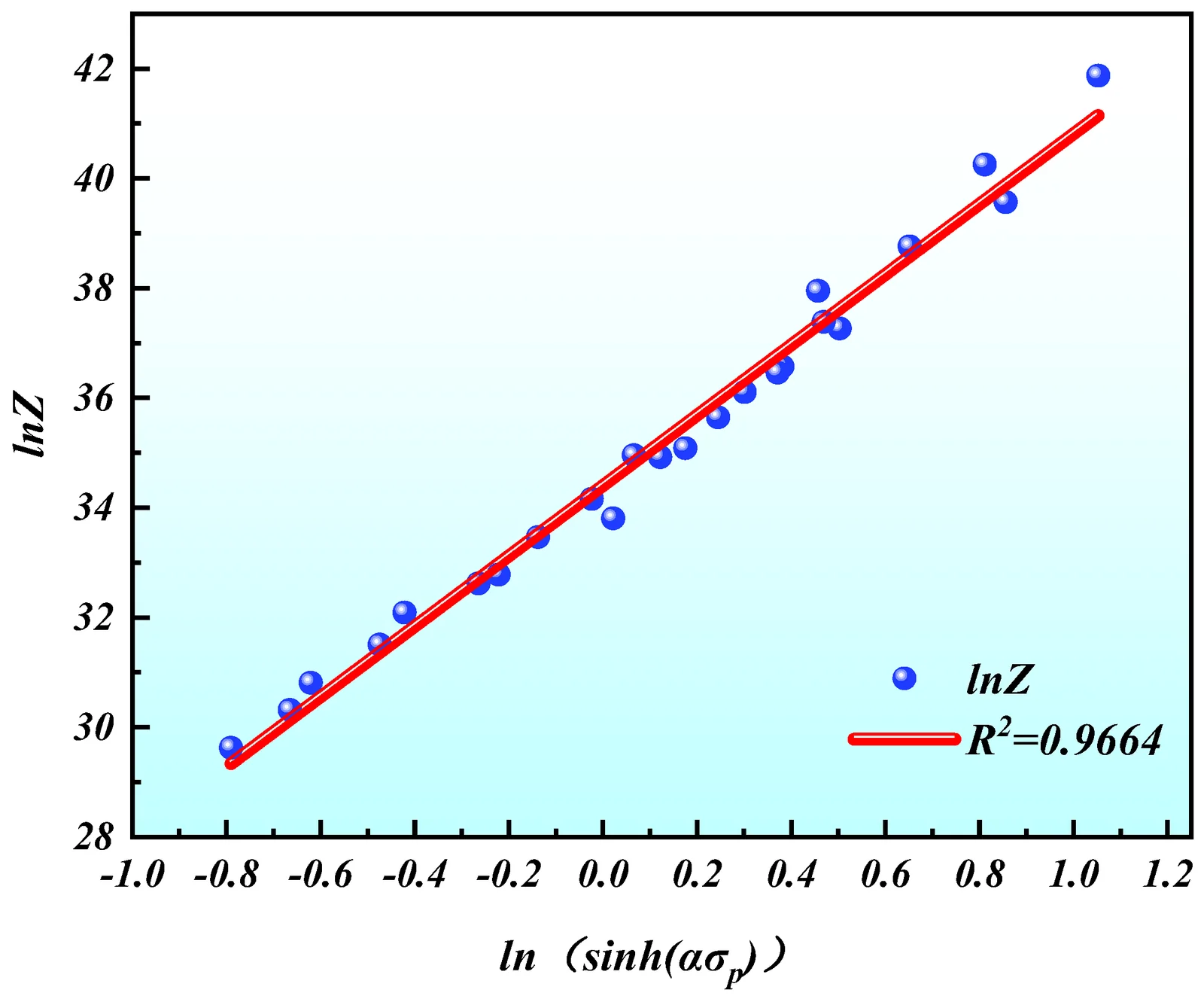
Relationship between peak stress and *Z*-parameters of Q420DR steel.

By combining Equation (1) and Equation (8), the quantitative link between the Z-parameter and peak stress can be constructed. As illustrated in Figure 6, the regression of ln*Z* against ln[sinh(ασp)] using peak-stress data from all investigated temperatures and strain rates gives a coefficient of determination of *R*^2^ = 0.9664. This value indicates a strong overall linear relationship in the transformed coordinates. The remaining deviations may reflect the use of a single set of material constants over the investigated temperature and strain rate range, together with experimental uncertainty. The slope of the fitted straight line is *n* = 6.505, and the intercept term gives ln*A* = 34.388.

All material constants of the Arrhenius-type constitutive model fitted from experimental peak-stress data are summarized as: *α* = 0.007724559 and *Q_d_* = 385.987 kJ·mol^−1^. The final constitutive formulas for Q420DR steel are expressed in Equations (11) and (12):

(11) $$\dot{\varepsilon }=e^{34.388}{\left[\sinh\left(0.0077245\sigma_{p}\right)\right]}^{6.50534}\exp\left(-385987/\mathrm{RT}\right)$$

(12) $$\sigma =129.46\ln\left\{{\left(Z/e^{34.388}\right)}^{1/6.50534}+{\left[{\left(Z/e^{34.388}\right)}^{2/6.50534}+1\right]}^{1/2}\right\}$$

To evaluate the predictive performance of the peak-stress-based Arrhenius model, the predicted peak stresses were calculated using the established model parameters for all 24 combinations of deformation temperature and strain rate. The predicted values were compared with the corresponding experimental peak stresses. The average absolute relative error (AARE) was used to quantify the prediction error:

(13) $$\mathrm{AARE}(\%)=\frac{100}{N}\sum_{i=1}^{N}\left|\frac{\sigma_{p,i}^{\mathrm{pred}}-\sigma_{p,i}^{\exp}}{\sigma_{p,i}^{\exp}}\right|$$

where *N* is the number of data points (*N* = 24), and $\sigma_{p,i}^{\mathrm{pred}}$ and $\sigma_{p,i}^{\exp}$ are the predicted and experimental peak stresses, respectively.

**Figure 7:**
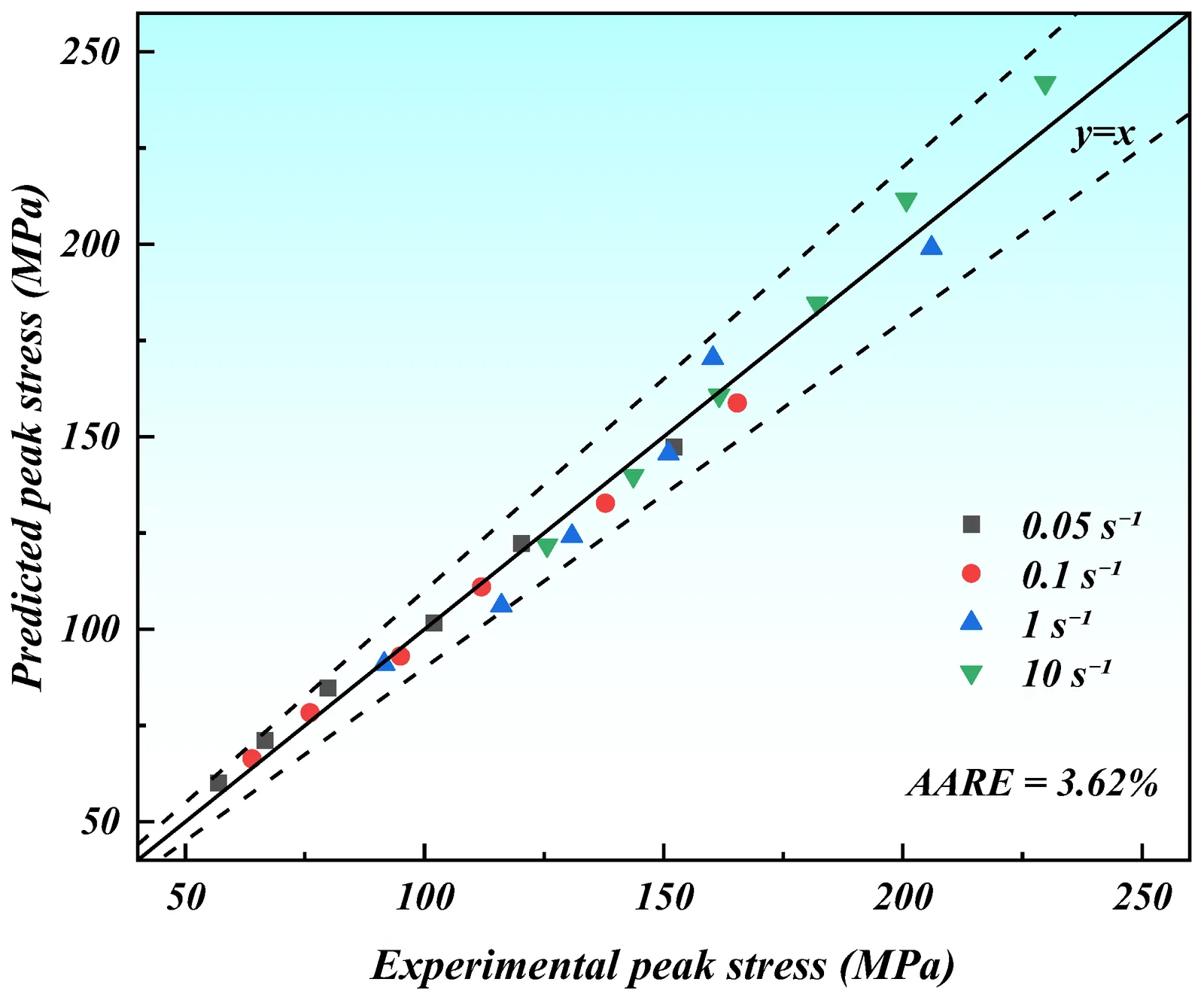
Comparison between predicted and experimental peak stresses of Q420DR steel under all investigated deformation conditions.

Figure 7 compares the predicted and experimental peak stresses over the investigated temperature range of 900–1150 °C and strain rate range of 0.05–10 s^−1^. The AARE is 3.62%, and all predictions fall within ±10% of the experimental values. The maximum absolute relative error is 8.57% at 1100 °C and 1 s^−1^, where the model underestimates the experimental peak stress. These results indicate that the established model reproduces the experimental peak stresses with good agreement within the investigated range. This assessment is based on the same dataset used to determine the model parameters.

### 3.3. Construction and Analysis of Hot-Processing Maps

Hot-processing mapping serves as a powerful analytical tool for probing high-temperature deformation responses and screening feasible thermomechanical processing windows for metallic materials. Its core objective lies in quantifying two critical indices, namely the power-dissipation efficiency *η* and flow-instability parameter *ξ*, across diverse temperature–strain rate combinations, so as to pinpoint the favorable parameter domain for hot forming. Such maps can reflect the intrinsic microstructural evolution tendency of alloys subjected to hot compression under varied thermomechanical loading.

A complete hot-processing map is composed of two sub-maps: the power-dissipation map and the plastic-flow-instability map. Joint interpretation of contour data from both sub-maps enables the identification of unstable deformation zones and the determination of suitable forming windows at different imposed strain magnitudes. In the present study, the power-dissipation contours and instability criteria contours were superimposed to construct integrated hot-processing maps for Q420DR steel.

The power-dissipation map adopted herein is built upon the Dynamic Material Model (DMM). From the perspective of DMM theory, there exists an energy-partition relationship between external input energy and energy consumption modes during hot deformation. A metallic workpiece under hot forming can be treated as a nonlinear energy-dissipative body. The total input power *P* is partitioned into two separate components: the power-dissipation content G and the complementary dissipation co-content *J*. Their mathematical relationship is given as:

(14) $$P=\sigma \dot{\varepsilon }=\int_{0}^{\sigma }\dot{\varepsilon }d\sigma +\int_{0}^{\dot{\varepsilon }}\sigma d\dot{\varepsilon }=G+J$$

In Equation (14), *G* represents the energy consumed by plastic deformation. Most of this portion of energy dissipates in the form of heat, while only a minor fraction is stored inside the material via crystalline defects. By contrast, the co-content *J* corresponds to energy consumed by microstructural evolution events, including dynamic softening, grain growth, dislocation rearrangement and phase transformation. For a fixed temperature and strain rate condition, the flow stress can be described by the power-law function:

(15) $$\sigma =k{\dot{\varepsilon }}^{m}$$

where *σ* is the flow stress; *k* is a material constant related to hot compression tests; $\dot{\varepsilon }$ is the strain rate; *m* is the strain rate sensitivity exponent, whose definition is given by the following equation:

(16) $$m=\left[\partial \ln\sigma /\partial \ln\dot{\varepsilon }\right]$$

Derived from Equations (14) and (15), the expression for *J* can be written as:

(17) $$G=\int_{0}^{\sigma }\dot{\varepsilon }\mathrm{d\sigma}=\int_{0}^{\dot{\varepsilon }}k{\dot{\varepsilon }}^{m}d\dot{\varepsilon }=\sigma \dot{\varepsilon }/\left(m+1\right)$$

(18) $$J=P-G=\sigma \dot{\varepsilon }-\sigma \dot{\varepsilon }/\left(m+1\right)=\left(m/m+1\right)\sigma \dot{\varepsilon }$$

*J*_max_ stands for the theoretical maximum complementary dissipation co-content. The magnitude of m is closely associated with defect-mediated deformation behaviors inside the material. An ideal dissipation status is achieved when *m* = 1, which gives the upper-limit value of *J*:

(19) $$J_{\max}=\sigma \dot{\varepsilon }/2$$

For real-world nonlinear dissipation scenarios, the dimensionless power-dissipation factor *η* is introduced to quantify the fraction of input energy consumed by microstructural evolution:

(20) *η* = *J*/*J*_max_ = 2*m*/*m* + 1

The dimensionless parameter *η* characterizes the ratio of actual microstructural-evolution-related dissipation to the ideal maximum dissipation under linear conditions. The magnitude of *η* can be adopted to assess the hot workability of Q420DR steel under different thermomechanical conditions. Higher η values signify more energy is diverted toward microstructural modification during deformation, which generally corresponds to superior hot formability. The power-dissipation map can be plotted using three variables: power-dissipation efficiency *η*, deformation temperature *T*, and strain rate $\dot{\varepsilon }$.

To generate power-dissipation contour maps, the strain rate-sensitivity exponent *m* needs to be calculated for each temperature–strain rate condition. For this purpose, experimental flow-stress and strain rate datasets are converted into logarithmic coordinates, followed by least-squares polynomial regression. Cubic polynomial fitting is widely recommended for this calculation to establish cubic spline functions. The value of *m* can then be extracted from the fitted regression curves.

Under given deformation temperatures, the correlation between ln*σ* and $\ln\dot{\varepsilon }$ can be expressed by the cubic polynomial function shown in Equation (21):

(21) $$\ln\sigma =a+b\ln\dot{\varepsilon }+c{\left(\ln\dot{\varepsilon }\right)}^{2}+d{\left(\ln\dot{\varepsilon }\right)}^{3}$$

where *a*, *b*, *c*, and *d* are regression coefficients of the cubic spline fitting function. Combined with the definition of strain rate-sensitivity exponent *m* can be expressed as:

(22) $$m=\partial \ln\sigma /\partial \ln\dot{\varepsilon }=b+2c\left(\ln\dot{\varepsilon }\right)+3d{\left(\ln\dot{\varepsilon }\right)}^{2}$$

**Figure 8:**
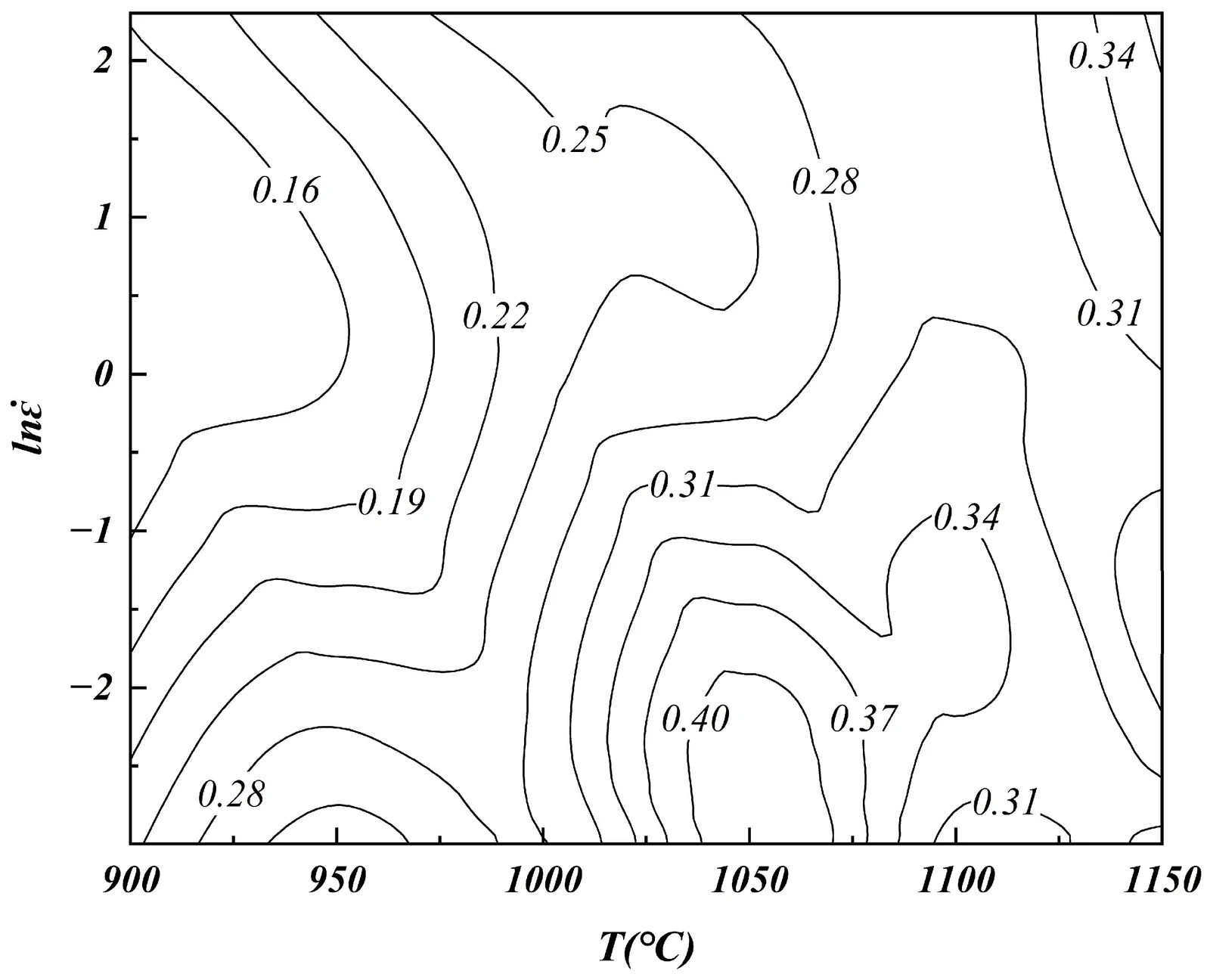
Power-dissipation map of Q420DR steel at a true strain of 0.6.

After computing *m* values for all experimental conditions via Equation (22), each *m* value is substituted into the formula for *η* to obtain corresponding power-dissipation-efficiency data. Deformation temperature is set as the abscissa, the natural logarithm of strain rate serves as the ordinate, and calculated *η* values are adopted for contour plotting. Figure 8 presents the power-dissipation map of Q420DR steel at a true strain of 0.6. The distribution of η depends on both deformation temperature and strain rate. Relatively high η values are observed at approximately 1050–1100 °C under low strain rate conditions, with contours reaching η = 0.40 near 1050 °C. These regions are further assessed using the flow-instability criterion to identify potentially favorable processing conditions.

In addition to power-dissipation maps, flow-instability maps constitute another essential component for constructing complete hot-processing maps. Plastic-flow localization may emerge in metallic alloys under certain hot-forming conditions, which makes instability evaluation necessary for practical process screening. Within the framework of the Dynamic Material Model (DMM), Ziegler originally proposed the fundamental instability criterion:

(23) $$\partial D/\partial \dot{\varepsilon }<D/\dot{\varepsilon }$$

where *D* denotes the dissipation function. Prasad further optimized this criterion by equating the dissipation co-content *J* to *D* and substituting it into Equation (23), which yields the modified instability relation:

(24) $$\partial \ln J/\partial \ln\dot{\varepsilon }<1$$

A natural logarithm transformation was performed on Equation (18), and the transformed result was incorporated into Equation (24). Subsequent mathematical rearrangement delivers the expression for the instability parameter *ξ*:

(25) $$\partial \ln J/\partial \ln\dot{\varepsilon }=\partial \ln\left(m/m+1\right)/\partial \ln\dot{\varepsilon }+\partial \ln\sigma /\partial \ln\dot{\varepsilon }+1<1$$

(26) $$\xi =\partial \ln J/\partial \ln\dot{\varepsilon }=\partial \ln\left(m/m+1\right)/\partial \ln\dot{\varepsilon }+m<0$$

By inserting the cubic-polynomial-form strain rate-sensitivity exponent *m* into the above formula, the final mathematical expression for the DMM-based plastic-flow-instability criterion can be obtained.

(27) $$\xi =\partial \ln\left(m/m+1\right)/\partial \ln\dot{\varepsilon }+m=2c+6d\ln\dot{\varepsilon }/m\left(m+1\right)+m<0$$

**Figure 9:**
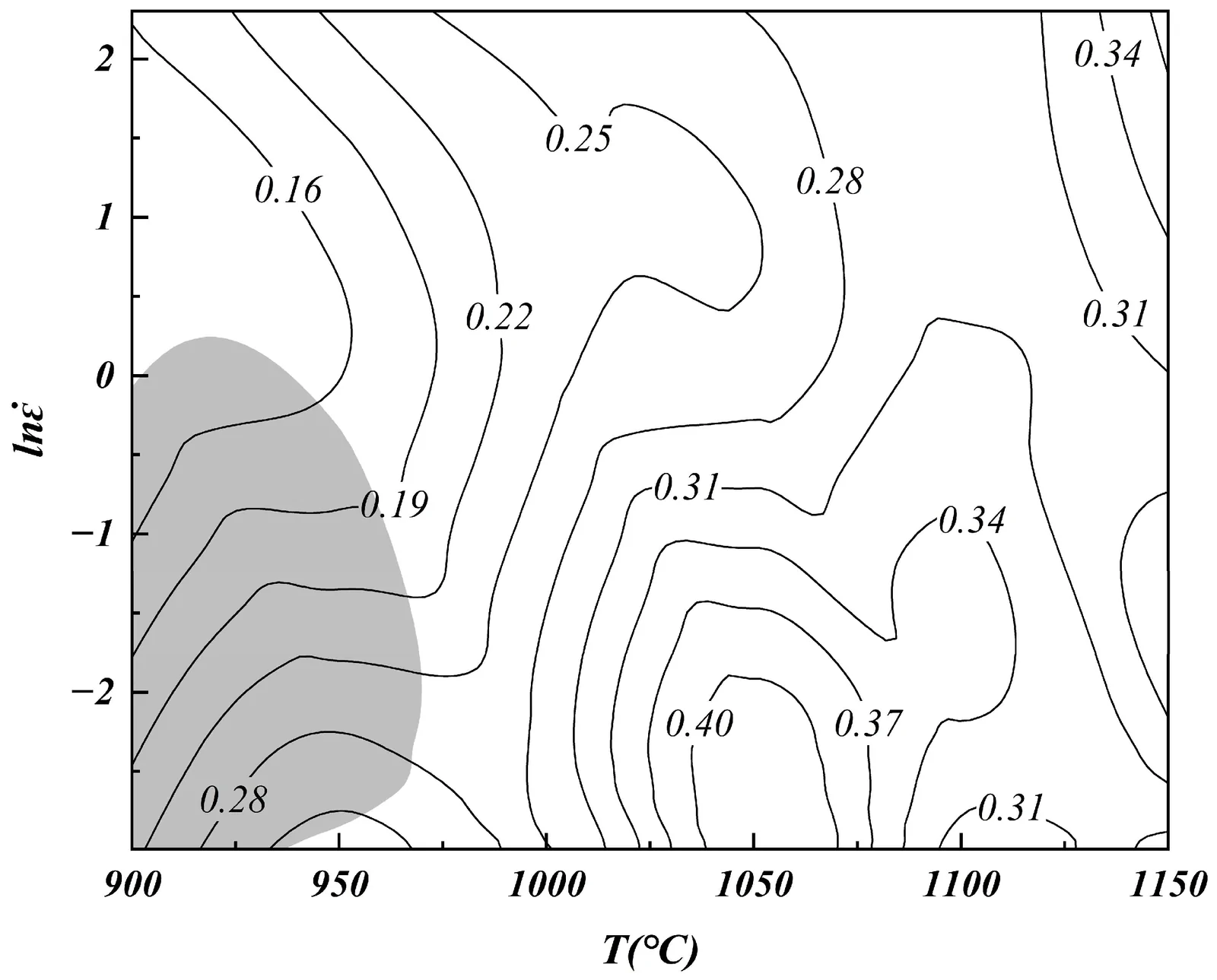
Hot-processing map of Q420DR steel at a true strain of 0.6.

The magnitude of the instability parameter *ξ* is governed by deformation temperature and strain rate. Negative *ξ* values correspond to the occurrence of plastic-flow instability. Regions where *ξ* remains below zero are defined as processing-instability domains, where non-uniform deformation is highly likely to take place. From a physical perspective, negative *ξ* implies that the material system fails to produce entropy at a rate sufficient to accommodate the imposed deformation rate. Under such circumstances, strain tends to concentrate into local bands, triggering flow-localization phenomena. According to the calculated distribution of *ξ* under different thermomechanical conditions, plastic-flow-instability maps can be plotted. Complete hot-processing maps are generated by overlaying power-dissipation contour maps with plastic-flow-instability maps, as illustrated in Figure 9.

At a given true strain, the instability parameter ξ varies with deformation temperature and strain rate. Within the adopted DMM criterion, ξ < 0 indicates predicted flow instability. Figure 9 combines the power-dissipation contours with the predicted instability region at a true strain of 0.6. The shaded region is mainly located at low temperatures and relatively low strain rates and extends to the lowest investigated strain rate of 0.05 s^−1^. Its boundary varies with temperature and strain rate and corresponds to ξ = 0. The high-efficiency region near 1050 °C at low strain rates lies outside the shaded region.

The predicted instability at low strain rates is not necessarily inconsistent with the DMM criterion, because ξ depends on both the strain rate sensitivity and its variation with strain rate, rather than on the absolute strain rate alone. A low-temperature, low-strain rate instability domain has also been reported for low-carbon Q690 V–N and V–N–Cr microalloyed high-strength steels at 850–866 °C and 0.01–0.024 s^−1^ [31]. For Q420DR steel, a possible explanation is that limited thermally activated restoration at relatively low temperatures may lead to spatially heterogeneous softening and nonuniform deformation, even when a longer deformation time is available.

**Figure 10:**
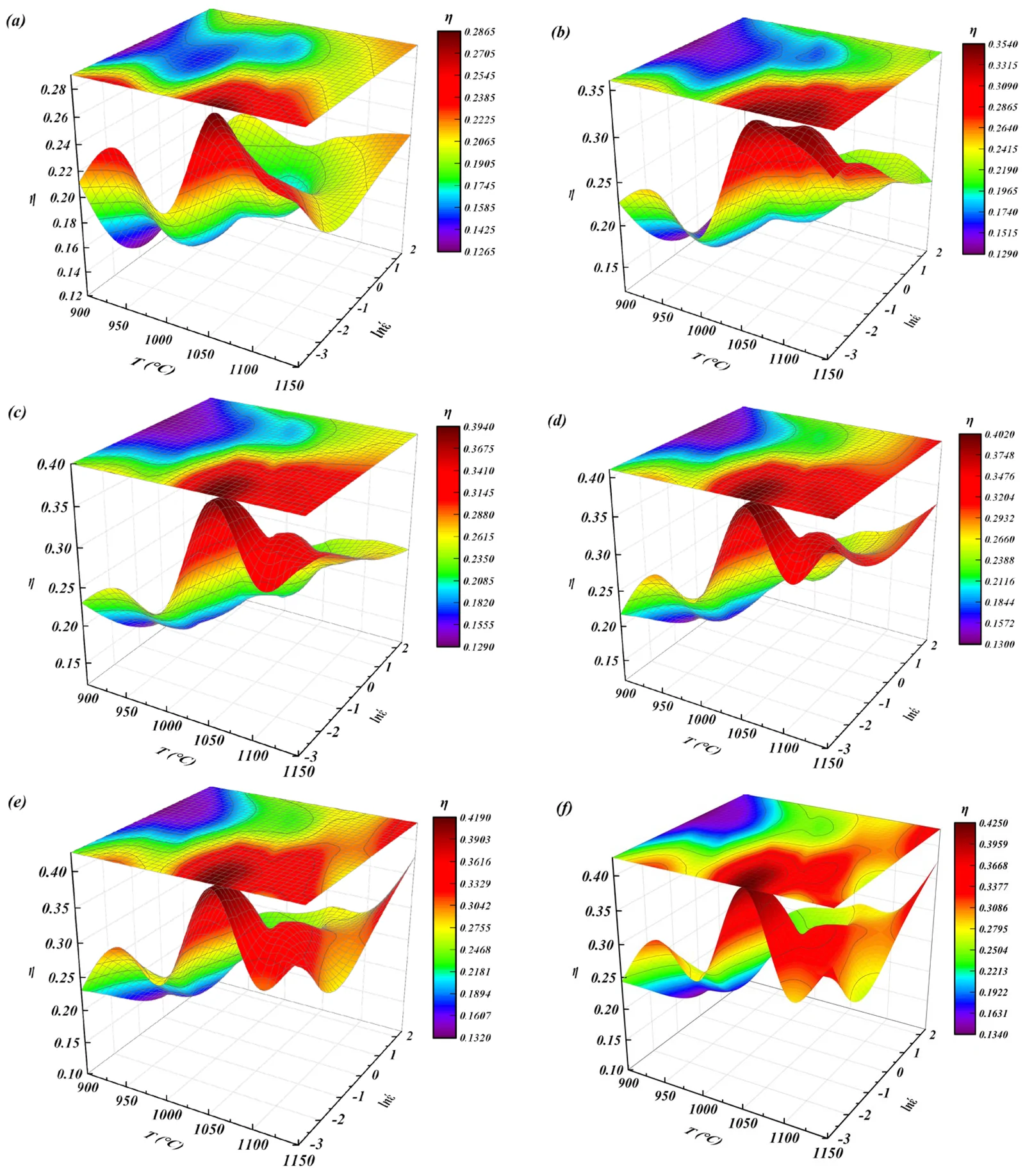
Power dissipation diagram of Q420DR steel at different strains. (**a**) *ε* = 0.1; (**b**) *ε* = 0.2; (**c**) *ε* = 0.3; (**d**) *ε* = 0.4; (**e**) *ε* = 0.5; (**f**) *ε* = 0.6.

The spatial distribution of power-dissipation efficiency *η* exhibits strong heterogeneity across the investigated parameter window, demonstrating that energy-dissipation behavior is highly sensitive to applied thermomechanical loading. In general, higher imposed true strain contributes to elevated *η* values, and high-efficiency dissipation zones become more prominent at larger strain levels. This trend indicates that a larger fraction of external deformation work is consumed by microstructural evolution events such as dynamic recovery and dynamic recrystallization as deformation proceeds. High-*η* domains are predominantly located at relatively high deformation temperatures coupled with low strain rates. Sufficient atomic diffusion and dislocation rearrangement can be activated under these circumstances, facilitating stable microstructural softening and improving the overall hot workability of Q420DR steel. The variation of power-dissipation efficiency *η* against temperature and strain rate at multiple true strains is presented in Figure 10.

**Figure 11:**
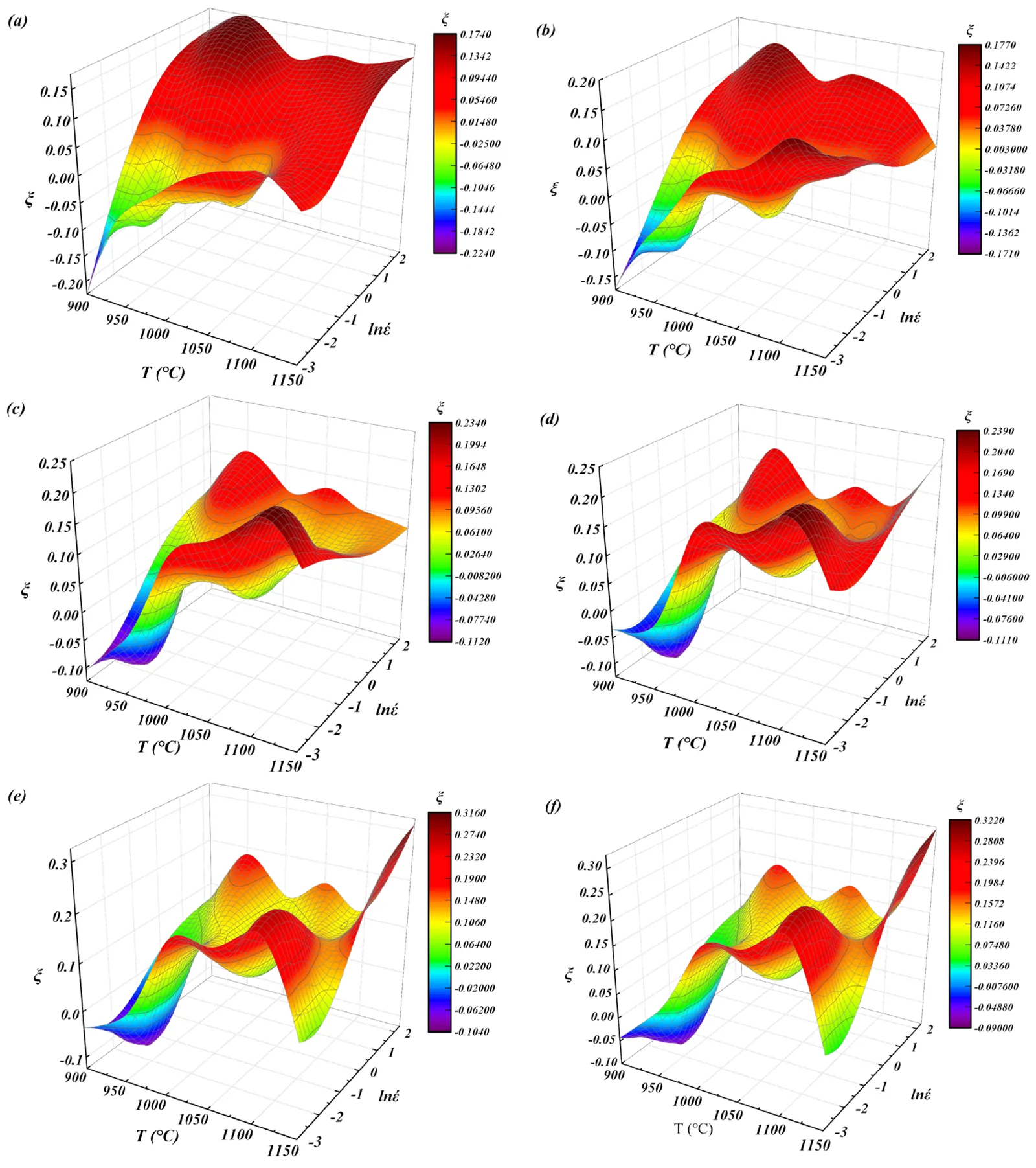
Instability of Q420DR steel at different strains. (**a**) *ε* = 0.1; (**b**) *ε* = 0.2; (**c**) *ε* = 0.3; (**d**) *ε* = 0.4; (**e**) *ε* = 0.5; (**f**) *ε* = 0.6.

The instability parameter ξ varies considerably with deformation temperature, strain rate and true strain, which reflects the high sensitivity of plastic-flow stability to thermomechanical conditions. In accordance with DMM theory, domains characterized by negative ξ values are regarded as unstable regions, where adverse defects including localized shear bands and inhomogeneous deformation may develop during hot deformation. For Q420DR steel, unstable zones mainly concentrate within the low-temperature regime. Insufficient thermal activation restricts the capacity of the microstructure to accommodate external deformation, raising the risk of flow localization. As true strain increases, the spatial distribution of ξ evolves gradually, and the area of stable domains with positive ξ expands correspondingly. This observation suggests that enhanced deformation strain improves the plastic-flow stability of Q420DR steel to a certain extent. The calculated ξ distributions for true strains ranging from 0.1 to 0.6 are summarized in Figure 11.

**Figure 12:**
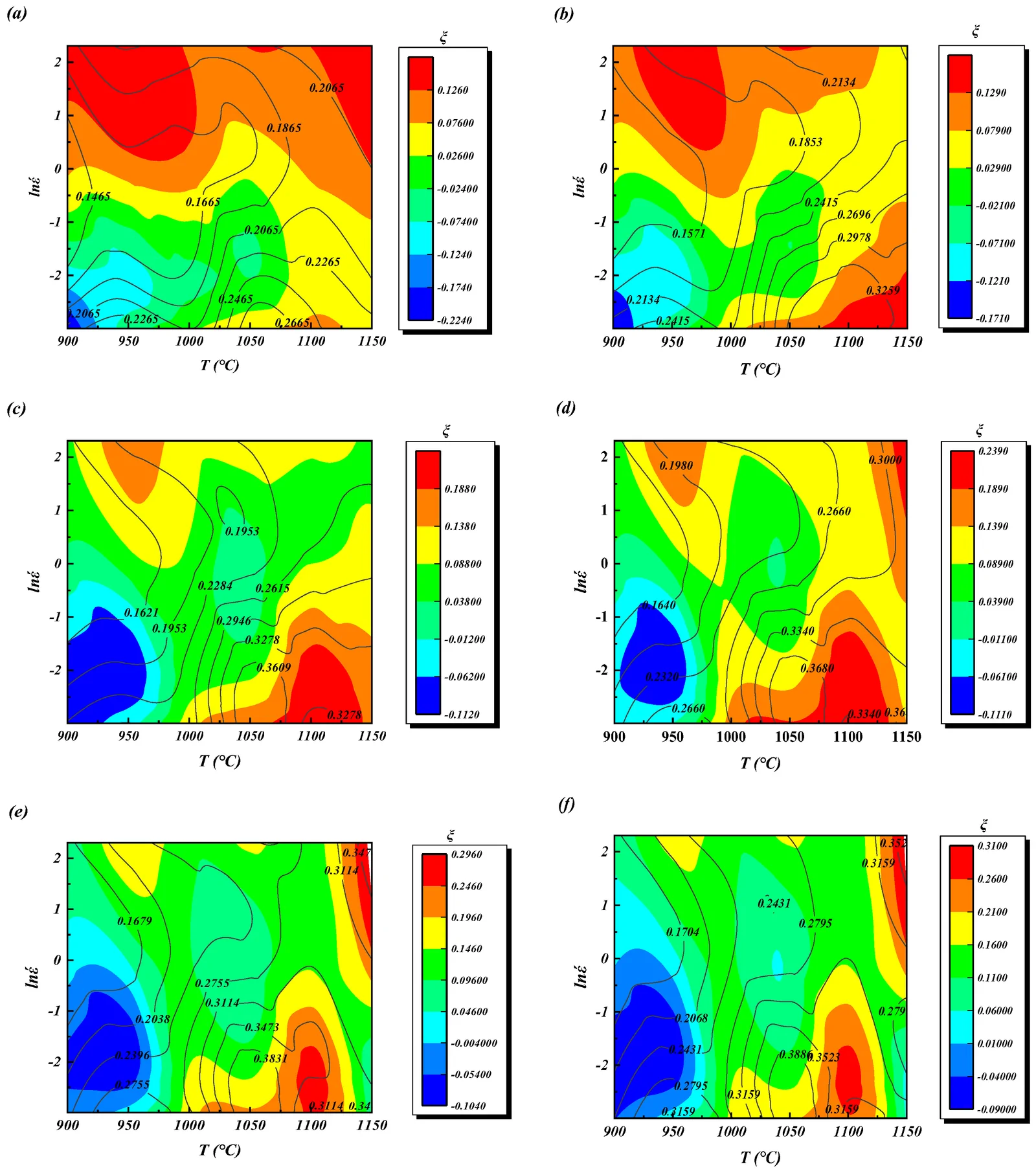
Hot processing maps of Q420DR steel. (**a**) 0.1 strain; (**b**) 0.2 strain; (**c**) 0.3 strain; (**d**) 0.4 strain; (**e**) 0.5 strain; (**f**) 0.6 strain.

Figure 12 shows a set of integrated hot-processing maps obtained by superimposing power-dissipation efficiency contours onto flow-instability maps at various true strains. Contour lines represent the magnitude of power-dissipation efficiency *η*, while colored zones mark regions with negative instability parameter *ξ*. Those colored instability domains should be avoided during practical hot forming, since deformation within such regions may induce non-uniform flow and localized damage. By contrast, regions combining relatively high η values with positive ξ values are identified as potentially favorable processing domains within the DMM framework. Based on this criterion, the processing map at a true strain of 0.6 indicates a candidate domain at approximately 1050–1100 °C under low strain rate conditions. This domain is a model-based prediction, and its boundaries and the predicted stable deformation behaviour require independent experimental validation.

Comparison of the processing maps at true strains of 0.1–0.6 indicates that the processing characteristics are strain-dependent, with the magnitude and spatial distribution of η and the predicted instability boundaries varying with strain. Therefore, the candidate domain identified at a true strain of 0.6 should not be assumed to remain unchanged throughout deformation. The selection of practical forming parameters should consider the evolution of the processing maps over the analysed strain range. These maps provide a DMM-based assessment of the hot workability of Q420DR steel. As direct microstructural evidence is not available in this study, the mechanisms associated with the identified domains, including possible dynamic recrystallization and localized deformation, remain to be confirmed by microstructural characterization.

## 4. Conclusions

(1) The flow behavior of Q420DR steel was sensitive to both deformation temperature and strain rate. Lower temperatures and higher strain rates increased deformation resistance, whereas higher temperatures and lower strain rates promoted more pronounced flow softening. These trends characterize the competing effects of work hardening and dynamic softening under the investigated compression conditions.
(2) A peak-stress-based Arrhenius-type hyperbolic-sine relationship was established to describe the dependence of peak flow stress on deformation temperature and strain rate. The apparent hot-deformation activation energy was 385.987 kJ·mol^−1^. This value represents the combined temperature sensitivity of the peak-stress response within the investigated range rather than the energy barrier of a single deformation mechanism. The resulting constitutive relationship is expressed as follows:
  $$\dot{\varepsilon }=e^{34.388}{\left[\sinh\left(0.0077245\sigma_{p}\right)\right]}^{6.50534}\exp\left(-385987/\mathrm{RT}\right)$$
(3) Processing maps constructed at true strains of 0.1–0.6 showed strain-dependent distributions of power-dissipation efficiency and predicted flow instability. At a true strain of 0.6, a candidate processing domain was identified at approximately 1050–1100 °C under low strain rate conditions, combining relatively high η values with positive ξ values. Predicted instability was mainly concentrated at low temperatures and relatively low strain rates. The candidate domain should not be regarded as invariant throughout deformation or as experimentally validated. Its boundaries and associated microstructural mechanisms require independent verification. The results provide preliminary guidance for single-pass hot compression; application to industrial rolling or multi-pass forging requires consideration of actual strain rates and thermal histories, together with process validation.

## Figures and Tables

**Table 1:** Chemical composition of Q420DR steel (wt.%).

Element	C	Si	S	P	Mn	Ni	Al	Cr	N	Nb	V	Ti	Mo	Cu	Fe
**Content**	0.15	0.35	0.001	0.01	1.62	0.49	0.03	0.02	0.003	0.037	0.054	0.016	0.003	0.13	Bal.

## Data Availability

The original contributions presented in this study are included in the article. Further inquiries can be directed to the corresponding authors.
